# Comparative Overview of Visuospatial Working Memory in Monkeys and Rats

**DOI:** 10.3389/fnsys.2016.00099

**Published:** 2016-12-16

**Authors:** Ken-Ichiro Tsutsui, Kei Oyama, Shinya Nakamura, Toshio Iijima

**Affiliations:** Division of Systems Neuroscience, Graduate School of Life Sciences, Tohoku UniversitySendai, Japan

**Keywords:** monkey, rat, lesion, single-unit recording, prefrontal

## Abstract

Neural mechanisms of working memory, particularly its visuospatial aspect, have long been studied in non-human primates. On the other hand, rodents are becoming more important in systems neuroscience, as many of the innovative research methods have become available for them. There has been a question on whether primates and rodents have similar neural backgrounds for working memory. In this article, we carried out a comparative overview of the neural mechanisms of visuospatial working memory in monkeys and rats. In monkeys, a number of lesion studies indicate that the brain region most responsible for visuospatial working memory is the ventral dorsolateral prefrontal cortex (vDLPFC), as the performance in the standard tests for visuospatial working memory, such as delayed response and delayed alternation tasks, are impaired by lesions in this region. Single-unit studies revealed a characteristic firing pattern in neurons in this area, a sustained delay activity. Further studies indicated that the information maintained in the working memory, such as cue location and response direction in a delayed response, is coded in the sustained delay activity. In rats, an area comparable to the monkey vDLPFC was found to be the dorsal part of the medial prefrontal cortex (mPFC), as the delayed alternation in a T-maze is impaired by its lesion. Recently, the sustained delay activity similar to that found in monkeys has been found in the dorsal mPFC of rats performing the delayed response task. Furthermore, anatomical studies indicate that the vDLPFC in monkeys and the dorsal mPFC in rats have much in common, such as that they are both the major targets of parieto-frontal projections. Thus lines of evidence indicate that in both monkeys and rodents, the PFC plays a critical role in working memory.

## Introduction

The term “working memory” refers to the cognitive ability to actively maintain and manipulate information that is behaviorally relevant. The concept of working memory extends far beyond that of short-term memory being a temporary storage of information, as working memory is assumed as a workplace for processing information. Baddeley and Hitch (1974) proposed a multi component model of human working memory, consisting of central executive, visuospatial sketchpad and phonological loop components, to which an episodic buffer as the fourth component was added later. Our current understanding of the neural mechanisms of working memory is mainly based on neuropsychological and electrophysiological experiments carried out on monkeys, many of which were focused on visuospatial functions. Recently, novel techniques derived from molecular biology have become common for rodents and started to provide further information concerning the role of specific receptors, cell types, and neural circuits. It is increasingly necessary to integrate the knowledge obtained from monkey and rodent experiments for a deeper understanding of the neural mechanisms linking molecular, cellular and systems levels. There is also a purely biological interest in comparing the neural background of common cognitive functions between different mammalian species. Here, we provide a comparative overview of visuospatial working memory in monkeys and rats on the systems level.

## Visuospatial Working Memory in Monkeys

### Neuropsychology—Lesion and Inactivation Studies

**Figure 1:**
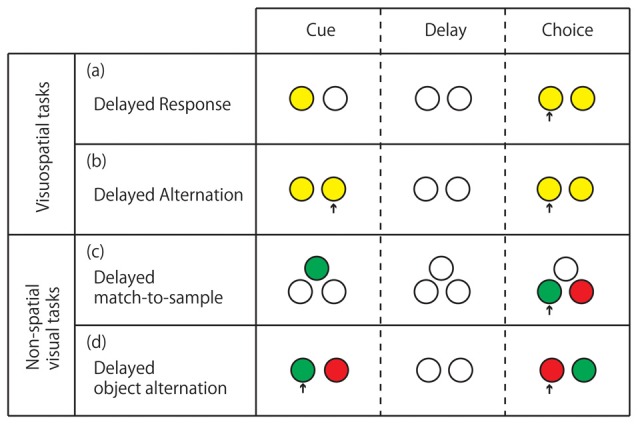
**Standard delay tasks for monkeys performed on computer-controlled push-button panel. (A)** Delayed response. The subject is required to memorize the location of the illuminated button and press it after the delay period when both buttons are illuminated. **(B)** Delayed alternation. The subject is required to alternate pressing the left and right buttons with intervening delays. The subject’s action in the previous trial serves as a cue in the present trial. **(C)** Delayed match-to-sample. The subject is required to memorize the color of the light illuminated at the cue period, and after the delay, press a button illuminated with the same color. **(D)** Delayed object alternation. The subject is required to alternate the choice between two colors with intervening delays. In **(C,D)** the color with which the two buttons are illuminated at the response period is randomized between trials. Arrows indicate the button pressed by the monkey.

For primates, various delay tasks have been used to study the neural background of working memory (for a review see Fuster, 2008). The standard tests for visuospatial working memory are “delayed response” and “delayed alternation” tasks, whereas those for nonspatial visual working memory are “delayed-match-to-sample” and “delayed object alternation” tasks (Figure 1). The prefrontal cortex (PFC) has been specified as the brain region responsible for visuospatial delay tasks, even well before the establishment of the concept of working memory. The first report of spatial delay task deficit due to a PFC lesion was made by Jacobsen (1936). Since then, a number of studies making smaller lesions within the PFC indicated that the ventral dorsolateral prefrontal cortex (vDLPFC), i.e., the area within and around the principal sulcus (Walker’s area 46), is the most critical region for visuospatial delay task performance (Mishkin, 1957; Gross, 1963; Goldman and Rosvold, 1970). From studies involving focal unilateral lesioning (Funahashi et al., 1993a) or induction of focal unilateral inactivation (Sawaguchi and Iba, 2001) in the vDLPFC of monkeys performing oculomotor delayed response tasks with eight possible target positions arranged in a circle at 45° intervals, and with 16 possible target positions of eight different directions and two different eccentricities respectively, the visuospatial working memory function of the vDLPFC was suggested to be topographically organized, with each hemisphere basically being responsible for the contralateral visual hemifield. It was concluded that the nature of the deficit induced by vDLPFC lesions or inactivation is based on the concept of “mnemonic scotoma”. Our recent study using low-frequency (1 Hz) repetitive transcranial magnetic stimulation (rTMS), with which we can temporarily inactivate the neural activity of the stimulated brain area, has shown that even in monkeys performing a delayed response task manually, unilateral inactivation of the vDLPFC yields visuospatial working memory deficits in the contralateral hemifield but not in the contralateral hand (Nakamura et al., 2014; Ogawa et al., 2015). In this temporal inactivation study using rTMS, monkeys were trained to manually perform a delayed response task with eight illuminable buttons arranged in a circle, similarly to the targets in oculomotor delayed response task in previous studies. The durations of the delay period (1.5, 4.5, 9, and 18 s) were randomized across trials. Low-frequency rTMS was applied either to the left or right vDLPFC before the daily task performance. During the daily session, left or right hand use was switched multiple times. Irrespective of the left or right hand use, the task performance was impaired in a delay-dependent manner only for targets contralateral to the stimulated hemisphere. This result may strongly support the idea that the memory coding in the vDLPFC is based on visuospatial but not on an effector-based coordinate.

Tsujimoto and Postle (2012) analyzed subjects’ responses in error trials in the oculomotor delayed response task with 16 possible target positions (arranged in eight directions and two eccentricities) and found that errors were made mostly by responding to the correct target position in the previous trial. On the basis of this finding, they proposed that the nature of deficits in delayed response tasks induced by vDLPFC lesions or inactivation is the susceptibility to proactive interference or perseveration rather than mnemonic scotoma. However, by analyzing the data from our study in which we examined the performance of a delayed response task while vDLPFC was inactivated by low-frequency rTMS (Nakamura et al., 2014; Ogawa et al., 2015), we found that most errors were made by responding to the target adjacent to the correct target in the current trial, suggesting the blurring of the topographically organized visuospatial working memory. We speculate that the inconsistency of results in those studies may be due to the difference of the severity of the visuospatial working memory deficit induced by experimental manipulations. A mild impairment of the vDLPFC function may induce the blurring of the memory of the current cue location, resulting in making errors by responding to a target adjacent to the correct one, whereas a severe impairment may induce almost complete disappearance of the memory of the current cue location, resulting in making errors by confusing the memory trace of the current and the previous cue locations. Thus we speculate that the result described by Tsujimoto and Postle (2012) does not contradict the idea of the topographical organization of visuospatial working memory in vDLPFC, but rather it may reflect the severity of deficits induced under their experimental conditions. This hypothesis should be tested in the future study by manipulating the severity of deficit by parametrically changing the amount of muscimol injection or the intensity of low frequency rTMS.

### Electrophysiology—Unit Recording Studies

**Figure 2:**
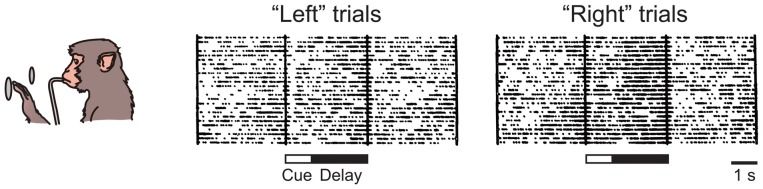
**Sustained delay activity recorded in the monkey ventral dorsolateral prefrontal cortex (vDLPFC) during performance of delayed response task.** This particular neuron showed higher activity during the delay in the “right” trial than in the “left” trial.

When Fuster and Alexander (1971) and Kubota and Niki (1971) independently recorded single-unit activity in monkeys performing delay tasks for the first time, they discovered a sustained increase in the firing rate of vDLPFC neurons during the delay period. Such an activity was considered to be the neuron-level correlate of short-term memory and was later reinterpreted as that of working memory. Niki (1974b) found that many of the neurons with a sustained delay activity exhibited different discharge rates depending on the location of the cue, e.g., a higher discharge rate for the “left” cue than for the “right” cue (Figure 2). It soon became an issue whether the differential activity codes the information of the cue presented or the action planned, i.e., the problem of retrospective sensory coding vs. prospective motor coding. By comparing the activity of a neuron in the standard delayed response task and in a task that requires a response to a direction different from that of the cue, Niki and Watanabe (1976) found that 70% of differential delay neurons coded the cue location, whereas the remaining 30% coded the response direction. Later, Funahashi et al. (1993b) confirmed the dominance of cue location coding over action direction coding in the vDLPFC by using oculomotor pro- and anti-saccade tasks. Thus, it was indicated that the majority of neurons in the vDLPFC are involved in the retrospective coding of visuospatial information, rather than prospective coding. By using an oculomotor delayed response task with eight possible target positions, Funahashi et al. (1989) found that the differential delay activity was finely tuned to a certain area in the visual field, normally on the contralateral hemifield. Together with their lesion and inactivation studies (Funahashi et al., 1993a; Sawaguchi and Iba, 2001), this suggests the topographic organization of the visuospatial working memory function in the vDLPFC. Although most attention has been paid to sustained delay activity since its discovery, transient activity for cue presentation, response execution, and reward delivery have also been reported from the early years of unit recording in the vDLPFC (Fuster, 1973; Kubota et al., 1974; Niki, 1974a; Niki and Watanabe, 1979). It has been discussed that transient activity during cue presentation is considered related to the encoding of information in working memory, whereas the transient activity after the delay period can be related to the extinction of working memory content, action execution, or evaluation of the outcome of one’s action (Fuster, 2008). More recently, it has been found that vDLPFC neurons show transient or sustained activity related to complicated visuospatial processes, such as route planning in a multistep maze (Mushiake et al., 2006) and perceptual categorization of arbitrarily distributed dots (Antzoulatos and Miller, 2011).

### Anatomy of Monkey PFC

**Figure 3:**
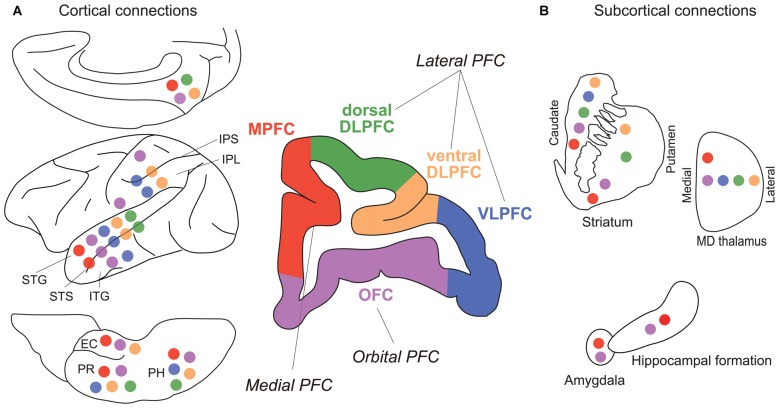
**Major fiber connections of monkey prefrontal cortex (PFC). (A)** Connections with other cortical areas. **(B)** Connections with subcortical areas. Colored circles represent connections with correspondingly colored areas of PFC (Green, dDLPFC; orange, vDLPFC; blue, VLPFC; purple, OFC; red, MPFC). Abbreviations: EC, entorhinal cortex; IPL, inferior parietal lobule; IPS, inferior parietal sulcus; ITG, inferior temporal gyrus; PH, parahippocampal cortex; PR, perirhinal cortex; STG, superior temporal gyrus; STS, superior temporal sulcus. (For references see Selemon and Goldman-Rakic, 1985; Ongur and Price, 2000; McFarland and Haber, 2002; Yeterian et al., 2012).

In addition to neuropsychology and electrophysiology, the anatomical connectivity, i.e., fiber projections, between brain regions provide key information for understanding brain functions on the systems level. The monkey PFC can be roughly subdivided into three areas: lateral, medial and orbital (Figure 3). As the lateral PFC is well interconnected with various sensory association and higher motor cortices, it may be mainly concerned with interaction with the external world, such as perception and recognition of external stimuli as well as planning and execution of motor actions. On the other hand, as the medial PFC is connected to medial temporal areas, such as the amygdala, the hippocampus, and their surrounding cortical areas, and the hypothalamus, it may be related to internal processes, such as long-term memory, emotion, and autonomic nervous system. The orbitofrontal cortex (OFC) seems to be specifically involved in reward, punishment and association learning, as it is connected to visual, olfactory and gustatory sensory areas as well as the amygdala, the hippocampus and their surrounding cortical areas, and the hypothalamus. Such an idea of broad functional segregation of the PFC is in accordance with the results of the default-mode analysis of data obtained by PET (Kojima et al., 2009) and fMRI (Mantini et al., 2011), and cortical network analysis of resting-state fMRI data (Hutchison and Everling, 2014). The lateral PFC can be further subdivided into vDLPFC (Walker’s area 46), ventrolateral PFC (VLPFC; Walker’s areas 12 and 45), and dorsal dorsolateral PFC (dDLPFC; lateral surface of Walker’s area 9 and 8B). The vDLPFC is mainly connected to various areas in the posterior parietal cortex (PPC), such as the superior and inferior parietal lobule (IPL), areas in the intraparietal sulcus (IPS), and the medial parietal (precuneus) cortex (Petrides and Pandya, 1984, 1999, 2006; Cavada and Goldman-Rakic, 1989). In contrast, the VLPFC is mainly connected with the temporal cortex, including the superior temporal cortex (STC), the inferior temporal cortex (ITC), and the areas in the superior temporal sulcus (STS; Webster et al., 1994; Borra et al., 2011; Saleem et al., 2014). The dDLPFC seems to function as an interface between the lateral and medial frontal cortices, having reciprocal connections to both the lateral and medial frontal cortices.

### Functional Organization of the Lateral PFC

On the basis of our current understanding of the anatomical connections of the PFC described above, it appears quite reasonable to consider that the lines of evidence from neuropsychological and electrophysiological studies indicate the critical involvement of the vDLPFC in visuospatial working memory. The PPC, which provides the major visual input to the vDLPFC, is the terminal region of the dorsal visual pathway. Lesions in this area cause poor performance in a “landmark test”, in which subjects are required to select a target closer to a landmark object, which reflects the deficit in the visuospatial guidance of action (Mishkin and Ungerleider, 1982). Strangely, however, some studies have shown that no deficit was observed in a delayed response task for inactivating the PPC (Fuster, 1995; Chafee and Goldman-Rakic, 2000), in which the visuospatial guidance of action is also necessary. Neuronal activity in the PPC during spatial delay tasks has been reported to be similar to that in the PFC, i.e., a large proportion of PPC neurons show a differential sustained activity during the delay period (Constantinidis and Steinmetz, 1996; Chafee and Goldman-Rakic, 1998; Qi et al., 2010). When tested using pro- and anti-saccade tasks, most of the PPC neurons were found to code the cue location (Gottlieb and Goldberg, 1999).

Beyond the critical involvement of the vDLPFC in visuospatial working memory, some studies have indicated the functional segregation of working memory within the PFC. Behaviorally, one can dissociate visuospatial and nonspatial object working memory by different types of delay tasks (Figure 1). Early lesion studies indicated that whereas the visuospatial working memory was most impaired by vDLPFC lesions (Mishkin, 1957; Gross, 1963; Goldman and Rosvold, 1970), the nonspatial visual working memory was most impaired by lesions in the VLPFC (Passingham, 1975; Mishkin and Manning, 1978). A single-unit study also showed the functional segregation between the vDLPFC and the VLPFC. That is, neurons related to visuospatial working memory were mainly found in the vDLPFC whereas those related to nonspatial visual object working memory were mainly found in the VLPFC (Wilson et al., 1993). Results of those neuropsychological and electrophysiological studies are in good agreement with anatomical connections. Namely, the vDLPFC is mainly connected to the PPC while the VLPFC is mainly connected to the ITC for visual input. However, the idea of the parallelism of the visuospatial and nonspatial working memories between vDLPFC and VLPFC may be an oversimplification (Rushworth and Owen, 1998). A number of single-unit recording studies showed that neurons related to nonspatial visual working memory were distributed not only in the VLPFC but also in the vDLPFC (Watanabe, 1986a; Quintana et al., 1988; Miller et al., 1996; Wallis and Miller, 2003b; Warden and Miller, 2010). Furthermore, other studies have indicated that the vDLPFC is concerned with abstract information beyond any sensory modality: a recent lesion study indicates that the vDLPFC is involved in working memory for abstract rule (Buckley et al., 2009). Additionally, there are a number of single-unit recording studies reporting sustained activity of neurons coding the abstract rule information (Wallis and Miller, 2003a; Yamada et al., 2010).

Unlike in the case of the vDLPFC or VLPFC, only a few studies examined the function of the dDLPFC specifically. Petrides (2000) showed by selective lesioning of the dDLPFC that the contribution of this area is critical when monkeys are required to maintain more than two items in their working memory at the same time.

## Visuospatial Working Memory in Rats

### Anatomy of Rat PFC

As the vDLPFC has been indicated as the most critical structure for visuospatial working memory in monkeys, a comparable area in rats would be the most promising candidate for having the same neural function. However, in rats, the anatomical definition of the PFC is not as clear as in monkeys (Preuss, 1995; Uylings et al., 2003). A classical definition of the PFC in primates is the existence of granular layer IV; therefore, the PFC has been referred to as the “frontal granular cortex”, but there is no such area in the rat frontal cortex. Using another definition, i.e., the projection from the thalamic nucleus medialis dorsalis (MD), we can define the PFC extending medially and ventrally in the anterior part of the cerebral cortex. For simplicity, we subdivide the PFC into two areas, medial and ventral. The medial and ventral areas of the PFC are referred to as the medial prefrontal cortex (mPFC) and OFC, respectively. The mPFC includes cytoarchitechtonically defined areas such as frontal area 2 (Fr2), dorsal anterior cingulate area (ACd), prelimbic (PL) and infralimbic (IL) areas. The OFC includes areas such as the medial, ventral, ventrolateral and lateral orbitofrontal cortices (MO, VO, VLO and VL, respectively).

**Figure 4:**
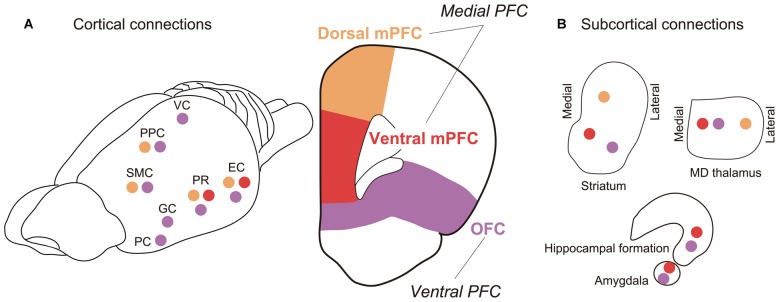
**Major fiber connections of rat PFC. (A)** Connections with other cortical areas. **(B)** Connections with subcortical areas. Colored circles represent connections with correspondingly colored areas of PFC (Orange, dmPFC; red, vmPFC; purple, OFC). Abbreviations: EC, entorhinal cortex; GC, gustatory cortex; PC, piriform cortex; PPC, posterior parietal cortex; PR, perirhinal cortex; SMC, sensorimotor cortex; VC, visual cortex. (For references see Reep et al., 1987, 1996; Vertes, 2004; Hoover and Vertes, 2007, 2011).

It appears that, according to the inter-regional connectivity, the mPFC can be further divided into two subareas: the dorsal mPFC, which corresponds to the cytoarchitechtonically defined areas Fr2 and ACd, and the ventral mPFC, which corresponds to PL and IL (Figure 4). Concerning the thalamo-cortical connectivity, the dorsal mPFC is reciprocally connected to the lateral part of the MD nucleus, whereas the ventral mPFC is reciprocally connected to the medial part of the MD nucleus (Uylings and van Eden, 1990). Concerning the cortico-cortical connectivity, the dorsal mPFC is reciprocally connected to the occipital, parietal and retrosplenial cortices, whereas the ventral mPFC is reciprocally connected to the rhinal cortex and amygdala (Ongur and Price, 2000; Uylings et al., 2003). The ventral mPFC can also be characterized as a medial prefrontal area that receives a heavy innervation from the hippocampus (Jay and Witter, 1991; Cenquizca and Swanson, 2007). These anatomical data suggest that the dorsal mPFC is the most likely candidate for the rat brain region comparable to the vDLPFC in the monkey brain, whereas the ventral mPFC in rats may be comparable to the mPFC in monkeys.

### Neuropsychology—Lesion Studies

**Figure 5:**
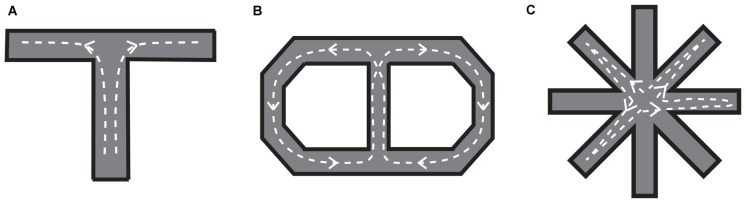
**Mazes for rats used to examine visuospatial working memory. (A)** T-maze. After a certain delay period, a barrier in front of the branching point is removed so that the rat can move into either the left or right arm. The left and right arms are alternatively baited in a series of trials. **(B)** Figure-eight maze. The rat is required to visit the left and right portions of the maze alternatively, always coming back to the central portion (it should run in the maze as indicated by the dotted lines with arrows). **(C)** Eight-arm radial maze. The subject is required to collect food that is baited at the end of each arm without re-entering the arms in which food has been already collected. To prevent subjects from developing a fixed sequence, four randomly selected arms are blocked until the rat goes into all of them then removed.

The widely used task to test visuospatial working memory in rats is delayed alternation in a T or Y maze (Figure 5). Eight-arm radial and figure-eight mazes are also common in testing the visuospatial working memory function. Kolb et al. (1974) tested for the first time whether visuospatial memory deficits can be observed in rats by lesioning a part of the frontal lobe using a delayed alternation task in a T-maze and a delayed response task in their original device. They found that the performance in those tasks was impaired by the mPFC lesion. Since then, a number of studies confirmed that a mPFC lesion leads to spatial working memory deficits detected as poor performance in the delayed alternation task in the T or Y maze (Larsen and Divac, 1978; Thomas and Brito, 1980; Eichenbaum et al., 1983; Wolf et al., 1987; Sánchez-Santed et al., 1997). From those studies, it appears that the impairment in the delayed alternation in the T or Y maze tends to be more severe when the lesion is limited to the dorsal part of the mPFC rather than when limited to the medial part of the mPFC. Kesner et al. (1996) dissociated the working memory for egocentric and allocentric spaces by using a six-arm modified plus maze and demonstrated that the egocentric working memory deficit (forgetting whether one has made a right or left turn before) is induced by a dorsal mPFC lesion, whereas the allocentric working memory deficit, forgetting which arm (place) one has been before, is induced by a ventral mPFC lesion. The difference in the spatial coordinate used in the dorsal mPFC and ventral mPFC may reflect the difference in the visuospatial information provided by their afferent connections. That is, the dorsal mPFC is mainly connected to the parietal cortex whereas the ventral mPFC is mainly connected to the hippocampus and its surrounding cortical areas (Jay and Witter, 1991; Ongur and Price, 2000; Uylings et al., 2003; Cenquizca and Swanson, 2007).

### Electrophysiology—Unit Recording Studies

Electrophysiological activities related to visuospatial working memory functions have not been extensively studied in rats as in monkeys, but there have been several studies that have shown neuronal activities in the rat mPFC which are presumably related to visuospatial working memory. Jung et al. (1998) recorded unit activity mainly in the mPFC during the performance of working memory tasks in an eight-arm radial maze and a figure-eight maze and reported a transient activity related to a specific timing in a trial or a specific place in the maze. Baeg et al. (2003) recorded unit activity in the mPFC during performance in a figure-eight maze and indicated that the left or right choice at the end of the central section of the maze can be predicted from the differential activity in the central section of the maze prior to the choice. Similarly, Yang et al. (2014) recorded unit activity in the mPFC during the performance of delayed alternation in a Y maze and found a choice-predicting differential activity during the delay period preceding the choice. Such a differential activity can be the retrospective coding of the choice in the previous trial or the prospective coding of the choice in the next trial. However, in both studies, the differential activity during the delay was transient in many neurons, and only a small population showed a sustained activity throughout the delay period. There has been a long debate on why the sustained delay activity can be only rarely found in the rat mPFC when performing delay tasks (Baeg et al., 2003; Yang et al., 2014).

One possible reason why previous studies failed to clearly show delay activity in rats is that the visuospatial working memory coding is different between monkeys and rats. It is the most straightforward idea that each of the neurons showing differential sustained delay activity carries information throughout the delay. On the other hand, Batuev et al. (1980) proposed a model in which ensembles of neurons showing transient activity in different timings can relay the information throughout the delay period. It is possible that in monkeys the working memory is coded in both ways, whereas in rats, mainly in the latter way. Another possibility is that the rarity of the sustained delay activity in rats is derived from task difference. During neuron recording, monkeys perform delay tasks manually as they sit in a primate chair with their head firmly fixed by a head-fixation device, whereas rats make locomotive movements without restrictions in a larger environment with respect to their body size. It is possible that in freely moving rats, prefrontal neurons, which fire transiently in relation to continuous sensory inputs and continuous motor planning and execution, overwhelm sustained delay neurons in number, whereas they remain silent in head-fixed monkeys. It is also possible that the sustained delay activity can be interrupted from certain sensory stimulation, which may shift the attention of a subject.

**Figure 6:**
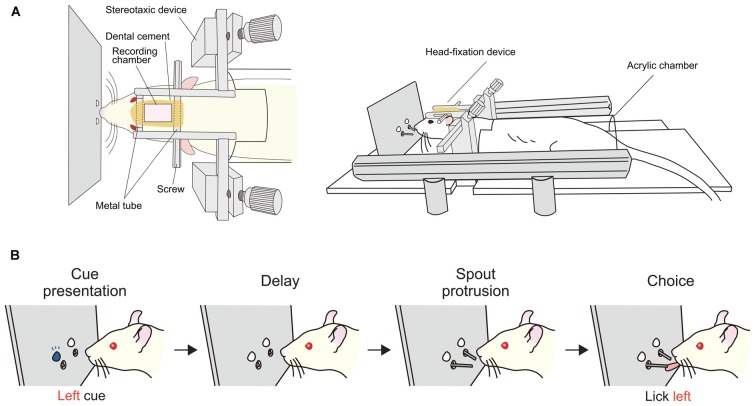
**Delayed response task for head-fixed rats. (A)** Apparatus used for experiments with head fixation (Left, top view; right, side view). A rat is laid in a prone position with its head fixed and body loosely restrained in a half-cylinder acrylic chamber. **(B)** Sequence of task events in a trial. At the beginning of a trial, an LED, either on the left or right, is illuminated for a short time then turned off. After a delay period, two spouts protrude towards the mouth of the rat. The correct response is to lick the same direction as the LED illuminated before the delay. The duration of the delay was typically 2 s for single-unit recording. Correct responses are rewarded with a drop of sucrose from the spout. Prior to the behavioral training, the head fixation device was implanted under anesthesia. After a period for recovery from the surgery, rats were habituated to the head-fixation condition by giving free reward from the spout. Then, the rats were trained in the delayed response task. As they performed about 300 to 400 trials per day, the correct rate gradually increased and reached over 80% in 2 or 3 weeks.

**Figure 7:**
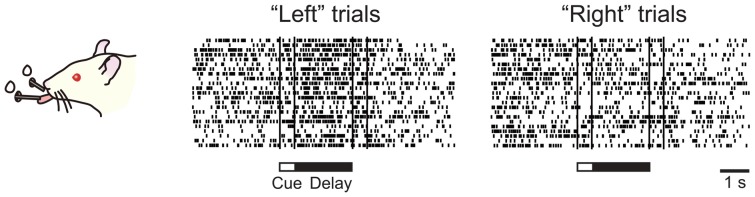
**Sustained delay activity recorded in the rat medial prefrontal cortex (mPFC) during performance of delayed response task.** This particular neuron showed higher activity during the delay in the “left” trial than in the “right” trial.

To address the second possibility, we recorded single-unit activity from the mPFC of head-fixed rats performing a delayed response task (Figure 6). We found a considerable number of neurons showing a sustained activity during the delay period, many of which were differential between “left” and “right” trials (Figure 7). Importantly, these sustained delay neurons appeared to be more densely distributed in the dorsal mPFC than in the ventral mPFC. We recorded from over 200 neurons from both areas and found that 17% of dorsal mPFC neurons showed differential sustained activity during the delay period of the delayed response task performance, whereas only 8% of ventral mPFC neurons did so. This result corresponds to the anatomical connectivity showing that the dorsal mPFC is the main target of parieto-frontal projections (Ongur and Price, 2000; Uylings et al., 2003), which may convey egocentric visuospatial information. To specify whether the recorded sustained delay activity was coding the location of the cue retrospectively or the direction of the movement prospectively, unit activity was recorded under the pro- and anti-response rules. Under the pro-response rule, a rat was required to lick the spout in the same direction as the cue illuminated before the delay period. Under the anti-response rule, the rat was required to lick the spout in the opposite direction from the cue illuminated before the delay period. The rule was altered every eight trials. Surprisingly, only less than 20% of all differential delay neurons coded the cue location, whereas the rest coded the response direction. This result is the opposite from those obtained from the monkey vDLPFC, where the vast majority of neurons coded the cue location retrospectively during delayed response performance. Further investigation is needed to specify whether the rat mPFC primarily codes the planned action or the result is dependent on the subjects’ strategy in performing the delayed response task.

## Limitations and Future Perspectives

In this article, we have provided a comparative overview of visuospatial working memory in monkeys and rats. Experimental neuropsychological studies over the years have indicated that the monkey vDLPFC plays critical roles in visuospatial working memory covering the peripersonal space, and probably the functionally comparable rat brain region may be the dorsal mPFC. Monkey electrophysiological studies have indicated that the sustained delay activity typically recorded in the vDLPFC may be the neural background of visuospatial working memory, and our recent study has found similar neuronal activity in the dorsal mPFC in rats. Anatomical studies indicate that the vDLPFC in monkeys and the dorsal mPFC in rats have much in common, such as that they are both the major targets of parieto-frontal projections. In summary of this review article, we conclude that to date accumulating evidence from anatomical, neuropsychological, and electrophysiological studies suggest the similarity between the monkey vDLPFC and rat dorsal mPFC in their roles in visuospatial working memory.

We should mention here the limitations of this review study. First, to keep the discussion well focused, we strictly limited the subject to visuospatial working memory in monkeys and rats, which resulted in focusing on a specific region of the PFC, the monkey vDLPFC and the rat dorsal mPFC. Much evidence from human neuropsychological and neuroimaging studies indicate that the human PFC is involved not only in visuospatial working memory but also in nonspatial working memory of various modalities, as well as many other aspects of cognitive and executive control functions (e.g., Owen et al., 1996; Koechlin et al., 1999; Olesen et al., 2004; for review, Stuss and Knight, 2002; Fuster, 2008; Passingham and Wise, 2012). Monkey electrophysiological studies have shown the neural correlates of various cognitive functions besides working memory within the PFC on the single-neuron level, such as response inhibition (Watanabe, 1986b), attentional control (Sakagami and Tsutsui, 1999; Lebedev et al., 2004), categorical recognition (Freedman et al., 2001; Antzoulatos and Miller, 2011; Tsutsui et al., 2016b), numerical recognition (Nieder et al., 2002), rule-based judgments (Wallis et al., 2001; Mansouri et al., 2006; Yamada et al., 2010), value-based decision making (Barraclough et al., 2004; Cai and Padoa-Schioppa, 2014; Tsutsui et al., 2016a), and complex action planning (Mushiake et al., 2006). We have no intention to insist that the function of the entire PFC can be solely explained by working memory, and indeed we admit that even the above mentioned list of PFC functions is not at all exhaustive. Nevertheless, working memory, i.e., the active maintenance and manipulation of information, may be the key element of any higher function that the PFC is responsible for, as we discuss in the last paragraph of this section. Second, we did not intend to make an exhaustive comparative study of monkeys and rats. Rather than comparing differences in various aspects of their physical and behavioral features, we focused on their common behavior, that is, they actively move around in the environment to explore and forage. Monkeys and rats use different types of senses to collect information from the environment; for example, what can be specific to rats may be whiskering and sniffing. Nevertheless, vision can be important in both monkeys and rats to recognize spatial information necessary to generate appropriate actions. In general, spatial information is supramodal, as it is established by combining information of different sensory modalities. Therefore, we consider that there can be many common aspects between different species for the neural coding of space. Indeed, by introducing the head-fixed experimental settings, we found neurons in the rat dorsal mPFC that code the location of sensory cues or the direction of an intended movement, similarly to what has been found in the monkey vDLPFC.

Here, we should also mention that the function of the rat frontal cortex is still under debate, with some researchers having views quite different from ours. Wise (2008) argued in his review comparing the frontal cortices of primates and rodents that there is no brain region in the rodent frontal cortex that is comparable to the primate PFC, referring to the conventional anatomical definition of the primate PFC as the frontal “granular” cortex, which is characterized by the prominence of granule cells in layer IV. However, if we refer to the inter-regional fiber projections, which constitute large-scale neural networks, instead of the cytoarchitecture mainly reflecting the features of a local neural network, there appears to be a common rule preserved between species: the dorsomedial, ventromedial, and orbital parts of the rat frontal cortex have similar cortico-subcortical and cortico-cortical projection patterns as the lateral, medial, and orbital parts of the monkey frontal cortex. As we have extensively reviewed in this article, neuropsychological and electrophysiological studies of monkeys and rats indicate that the monkey vDLPFC and rat dorsal mPFC appear to play a critical role in visuospatial working memory. By citing several monkey neuropsychological and electrophysiological studies, Wise (2008) further argued that the functional characteristics of the granular cortex in primates is not working memory, or the temporary storage of behaviorally relevant information, but the storage of “knowledge” that guides nonroutine behavior, such as rules and strategies. Indeed we admit that the rat PFC is not a replica-in-miniature of the monkey PFC, just as the monkey PFC is not that of the human PFC. Behavioral flexibility, which may be a manifestation of the PFC function, is more prominent in monkeys than in rats, and in humans than in monkeys. However, if the rule- or strategy-based behavior was specifically associated with the granular frontal cortex, the logical expectation is that rodents that lack the granular frontal cortex should not exhibit rule- or strategy-dependent behavior. In our studies, however, the rats learned to switch between pro- and anti-licking delayed responses as frequently as every eight trials. We consider that the notion that rodents do not have any PFC at all may be an underestimation of the capacity of the rodent frontal cortex function.

For the next step of the comparative study of the visuospatial working memory, we consider that it is important to investigate the flow of information in a large-scale network in both monkeys and rats. For such a purpose, we can benefit from recent progress in analytical methods and computing power. By the network information flow analysis of various forms of neural data, not only PET and fMRI images, but also simultaneously recorded electrocorticogram (ECoG), local field potential (LFP), and single-/multiple-unit activities throughout multiple brain regions, we may reveal how different brain areas work in harmony and how information is processed throughout the neural network. Furthermore, new techniques, such as optogenetics and TMS, that enable the event-related manipulation of local neural activity during task performance would be useful to test the validity of a network information flow model. The proposed inter-cellular mechanism of sustained delay activity is a reverberating neural circuit. The simplest of such circuit is reciprocally connected to excitatory neurons. Empirically, both the monkey vDLPFC (Petrides and Pandya, 1984, 1999, 2006; Cavada and Goldman-Rakic, 1989) and rat dorsal mPFC (Ongur and Price, 2000; Uylings et al., 2003) are reciprocally connected to the PPC. They also form thalamo-cortical and cortico-striato-thalamo-cortical circuits, as the other frontal regions do (Alexander et al., 1986). It is quite possible that the closed-loop reverberating circuit is included in those inter-regional projections. We will be able to test these hypotheses both in the monkey and rat brains by applying the network information flow analysis of various kinds of neural data simultaneously obtained throughout the brain.

Visuospatial working memory is the most well-studied function of the PFC and has been a central topic of PFC research for a long time. However, it is only a part of a vast variety of PFC functions. Dysfunction of the PFC leads to deficits in various cognitive abilities, such as visuospatial and object working memory and attention, inhibitory control of movement, motivational and emotional regulation, prospective inference, behavioral planning, and decision making (Stuss and Knight, 2002; Fuster, 2008; Passingham and Wise, 2012). Nonetheless, we believe that the investigation of the visuospatial working memory function using standard delay tasks would lead us to a fundamental understanding of the PFC function in general. One important aspect of the visuospatial working memory is that it can encode and extinguish information whenever necessary. Not only immediate encoding of information but also immediate clearance of the memory buffer is essential for avoiding confusion regarding the memorized information between trials. Indeed, proactive interference, the interference of the past memory over the new memory, occurs owing to PFC damage. Another important aspect is the conversion of information, such as from visual to motor, in the case of a delayed response. The PFC is capable of switching between different conversion rules immediately, such as from the pro- to anti-response rule or vice versa. In humans, such function can be examined using the Wisconsin Card Sorting Test, which has been a standard neurological procedure to test the PFC function. Immediate encoding, extinction and multiple conversion of information seem to be the key features of the PFC, which cannot be observed in other cortical regions, and may be the biological background of cognitive thought processes. These views are still at the level of working hypotheses, but we consider that this kind of reductionist attitude would be of much importance when investigating the function of the PFC, as we normally tend to end up adding a new item to a long-lasting list of PFC functions after conducting a new study. In addition, together with studies directly testing the working hypotheses, studies showing what kind of function a certain part of the PFC is “not” involved (e.g., Baxter et al., 2008; Minamimoto et al., 2010) can sometimes be more informative than so-called “positive” reports that further extend the list of PFC functions.

## Ethics Statement

Animal Care and Use Committee of the Tohoku University Environmental & Safety Committee.

## Author Contributions

K-IT, KO, SN and TI wrote the manuscript while K-IT took a major role.

## Conflict of Interest Statement

The authors declare that the research was conducted in the absence of any commercial or financial relationships that could be construed as a potential conflict of interest.
